# Parenchymal Extinction Mimicking Hepatocellular Carcinoma in a Patient with Chronic Hepatitis B-Related Liver Cirrhosis

**DOI:** 10.3390/diagnostics11071171

**Published:** 2021-06-28

**Authors:** Min Kyu Kang, Joon Hyuk Choi

**Affiliations:** 1 Department of Internal Medicine, College of Medicine, Yeungnam University, 170 Hyunchungro, Namgu, Daegu 42415, Korea; 2 Department of Pathology, College of Medicine, Yeungnam University, 170 Hyunchungro, Namgu, Daegu 42415, Korea; joonhyukchoi@ynu.ac.kr

**Keywords:** parenchymal extinction, hepatocellular carcinoma, alpha-fetoprotein, dynamic T1-weighted MRI scan

## Abstract

Parenchymal extinction is characterized by the irreversible loss of hepatocytes and their eventual replacement by fibrous tissue, along with the alteration of the sinusoidal architecture and the obstruction of the small portal and hepatic veins. In clinical practice, radiologic modalities are not sufficient for differentiating between parenchymal extinction and hepatocellular carcinoma in patients with advanced fibrosis or cirrhosis. Herein, we present a case of parenchymal extinction mimicking hepatocellular carcinoma in a patient with chronic hepatitis B-related liver cirrhosis.

## 1. Introduction

Parenchymal extinction is defined as the loss of contiguous hepatocytes in a pathologic examination [1]. Considering that the loss of hepatocytes is related to adjacent tissue hypoxia, parenchymal extinction has been newly defined as a region of focal loss of hepatocytes with adjacent microvascular structures [2]. Hepatocellular carcinoma (HCC), one of the most common malignant tumors with the third-highest cancer-related mortality worldwide, is closely associated with liver cirrhosis caused by a variety of underlying etiologies, including chronic viral hepatitis B and C, and alcohol intake [3,4]. The diagnosis of HCC is based on radiologic modalities, including computed tomography and/or magnetic resonance imaging (MRI), usually without histologic confirmation because of the risk of lethal complications including needle track seeding, bleeding, and death [5,6]. Therefore, it is difficult to differentiate between parenchymal extinction and HCC in patients with advanced fibrosis or cirrhosis in clinical practice. We present a case of parenchymal extinction mimicking HCC in a patient with chronic hepatitis B-related liver cirrhosis.

## 2. Case Presentation

A 47-year-old man, who was a farmer, with chronic hepatitis B infection and Child-Pugh class A cirrhosis, was referred to the Department of Internal Medicine with a markedly elevated alpha-fetoprotein (AFP) level and a hypoechoic hepatic lesion with a diameter of approximately 3 cm, observed with abdominal ultrasonography. He had been treated with entecavir and adefovir for 5 years at a local clinic but had voluntarily stopped taking antiviral agents a year ago. His initial laboratory findings were as follows: aspartate aminotransferase (AST) level, 170 IU/L; alanine aminotransferase (ALT) level, 278 IU/L; total bilirubin level, 1.2 mg/dL; albumin level, 3.69 g/dL; platelet count, 103 × 10^3^/μL; prothrombin time (PT), 14.8 s; and AFP level, 585.2 ng/mL (normal range: 0–7 ng/mL). After confirming viral breakthrough with the presence of 2.8 × 10^7^ IU/mL hepatitis B virus (HBV) DNA, and the drug-resistant mutations L180M and M204I, we switched the antiviral treatment to tenofovir disoproxil fumarate monotherapy.

**Figure 1:**
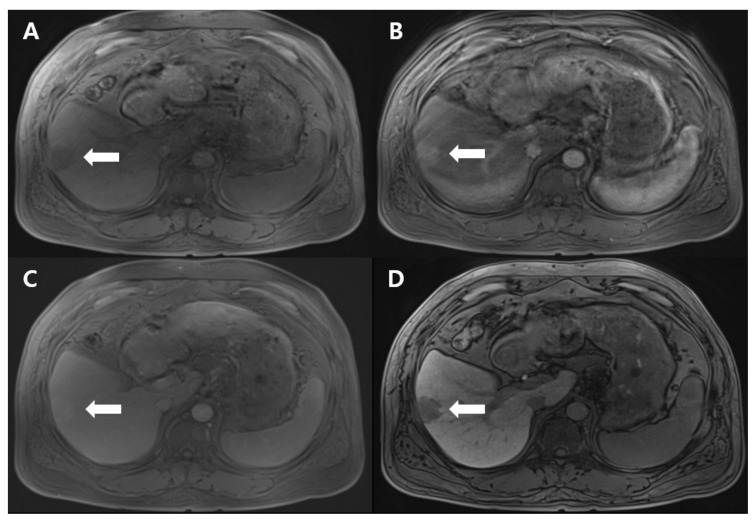
T1-weighted magnetic resonance imaging reveals an approximately 3-cm-sized, lobulated, hypointense mass in the pre-contrast phase (**A**), a hyperintense mass in the arterial phase (**B**), an isointense mass in the delayed phase (**C**), and a hypointense mass in the hepatobiliary phase (**D**), at segment 6. The lesion is indicated by white arrows in all phases.

Dynamic T1-weighted MRI revealed a 3-cm-sized, lobulated mass at segment 6, demonstrating hyperintensity in the arterial phase, isointensity in the delayed phase, and hypointensity in the hepatobiliary phase, suggesting the possibility of HCC (Figure 1). Due to the elevated AFP level and the peripheral location of the mass, tumor resection was performed instead of a biopsy.

**Figure 2:**
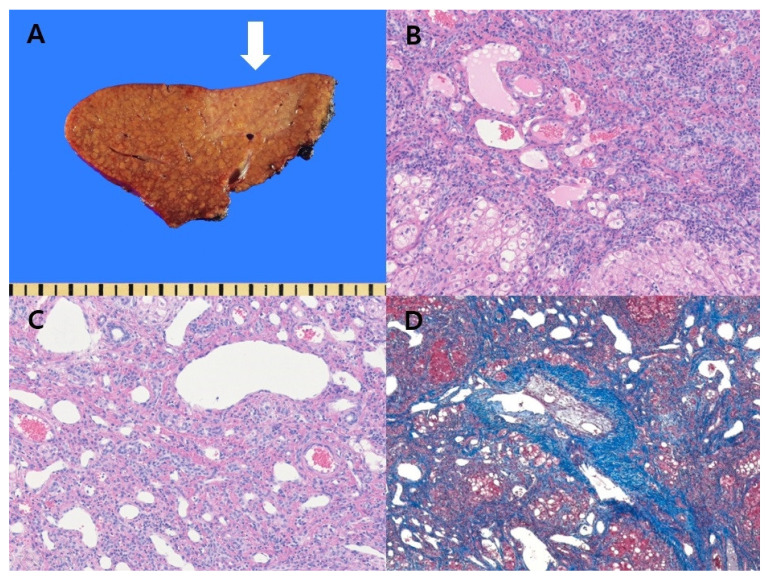
(**A**) Gross finding of the tumor. Grossly, an approximately 3-cm-sized, bright yellow mass-like lesion revealing ill-defined margins in the cirrhotic liver (white arrow); (**B**,**C**) Microscopic findings reveal focal loss of hepatocytes with increased capillary-sized vessel dilatation (**B**,**C**: hematoxylin and eosin staining, ×40, ×100, respectively); (**D**) Abundant fibrous septa with dilated capillary vessels are seen. (**D**: Trichrome staining ×40).

Histological examination of the resected mass revealed the loss of focal hepatocytes with increased capillary-sized vessels and fibrotic tissue in the cirrhotic liver, consistent with findings of parenchymal extinction (Figure 2). Some blood vessels in the lesion were partially occluded by intimal thickening. To exclude the presence of HCC, immunohistochemical staining was performed. The hepatocyte lobules in the lesion were negative for glypican-3, which is a diagnostic marker for HCC.

**Figure 3:**
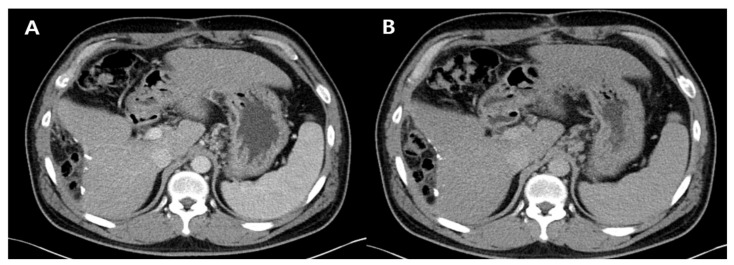
Follow-up dynamic computed tomography scan after 1 year. Multiple surgical clips at the previously resected margin are seen without new lesions: arterial phase (**A**) and delayed phase (**B**).

After approximately 1 year, the patient’s laboratory and radiological findings were normalized (Figure 3). His follow-up laboratory tests were as follows: AST level, 20 IU/L; ALT level, 31 IU/L; total bilirubin level, 0.63 mg/dL; albumin level, 3.56 g/dL; platelet count, 127 × 10^3^/μL; PT, 11.2 s; and AFP level, 5.1 ng/mL. The repeat HBV DNA was undetectable.

## 3. Discussion

We presented a case of parenchymal extinction mimicking HCC in a patient with chronic hepatitis B-related liver cirrhosis. Wanless et al. defined parenchymal extinction as the irreversible loss of hepatocytes and their replacement by fibrous tissue [2,7]. The putative mechanism of parenchymal extinction is linked to hepatic necro-inflammation due to chronic injury, including viral hepatitis and alcoholic liver disease, which leads to micro-infarcts by occlusive thrombi [8,9]. In patients with cirrhosis, micro-thrombosis of the sinusoids and the obliteration of the small hepatic vessels leads to tissue ischemia and hepatocyte apoptosis [10]. Contiguous hepatocyte ischemia, tissue necrosis, and approximation of the adjacent portal and hepatic veins are typically observed [11]. Eventually, the parenchymal extinction lesion is replaced by fibrotic tissue. In our case, although no definite micro-infarcts with occlusive thrombi were observed, parenchymal extinction was diagnosed by the loss of hepatocytes and the presence of fibrotic tissue with abundant inflammatory cell infiltration and absent hepatocytes and cancer cells. Additionally, the diagnosis was confirmed based on the peripheral wedge resection of the lesion. This allowed us to effectively treat the patient. 

The limitations of our case are as follows. First, we could not perform another radiologic technique, such as contrast-enhanced ultrasonography (CEUS), for the diagnosis of HCC. On CEUS, arterial phase hyper-enhancement with mild late-onset (>60 s) washout is pathognomonic of HCC [12,13]. In a recent prospective multicenter study, CEUS had a specificity of 92.9% compared to 83.2% for MRI in diagnosing 1- to 2-cm HCCs [14]. In addition, when MRI is inconclusive, CEUS, as an additional imaging modality, has the highest sensitivity and specificity among other modalities for 2- to 3-cm HCCs [14].

Second, we could not perform a biopsy of the suspected HCC lesion because of the possibility of needle track seeding. Instead, wedge resection was performed to simultaneously diagnose and treat the peripheral lesion. In previous studies, needle track seeding is shown as manageable by excision or radiation therapy and does not affect the clinical outcome or overall survival [15,16]. Therefore, it is now widely accepted that diagnostic liver biopsies of suspected HCC lesions are safe [17].

## 4. Conclusions

A target biopsy is required for the diagnosis of the lesion suspected to be HCC.

## 5. Highlights

Parenchymal extinction can be difficult to differentiate from HCC in patients with cirrhosis.

When the diagnosis of HCC is uncertain using one radiologic modality, alternative modalities should be considered.

Diagnostic liver biopsy of suspected lesions should be considered prior to surgery, when feasible.

## Data Availability

The data that support the findings of this study are available from the corresponding author (M.K.K.) upon reasonable request.
